# The Differential Contribution of Macular Pigments and Foveal Anatomy to the Perception of Maxwell’s Spot and Haidinger’s Brushes

**DOI:** 10.3390/vision7010011

**Published:** 2023-02-06

**Authors:** Gary P. Misson, Rebekka Heitmar, Richard Armstrong, Stephen J. Anderson

**Affiliations:** 1 School of Optometry, College of Health & Life Sciences, Aston University, Birmingham B4 7ET, UK; 2 Department of Optometry and Vision Sciences, Centre for Vision across the Life Span, University of Huddersfield, Queensgate, Huddersfield HD1 3DH, UK

**Keywords:** entoptic phenomena, macula, macular pigment, polarized light, central vision, fovea, Maxwell’s spot, Haidinger’s brushes

## Abstract

The relationship of macular pigments and foveal anatomy to the perception of Maxwell’s spot (MS) and Haidinger’s brushes (HB) entoptic phenomena were investigated. Dual-wavelength-autofluorescence and OCT were used to define macular pigment density and foveal anatomy in 52 eyes. MS was generated by alternating unpolarized red/blue and red/green uniform field illumination. HB was generated by alternating the linear polarization axis of a uniform blue field. In Experiment 1, horizontal widths of MS and HB were measured using a micrometer system and compared with macular pigment densities and OCT-defined morphometry. MS radius (mean 1.4°) was significantly less than HB radius (mean 1.6°), with the spatial extent of both phenomena falling between the boundaries of the foveola and foveal pit. Multiple regression showed MS and HB radii to be significantly associated with the macular pigment spatial profile radius. HB radius, but not MS radius, was also significantly associated with foveolar morphometry. Experiment 2 compared perceptual profiles of MS with macular pigment distribution patterns and demonstrated close agreement. The size and appearance of MS is a direct indicator of macular pigment density and distribution. Measures of HB radii are less specific, with dependence on both macular pigment density and foveal structure.

## 1. Introduction

Normally sighted individuals can perceive a short-lived darkened spot at the point of fixation while viewing a plain white surface through a dichroic filter transmitting a mixture of long- and short-wavelength lights [1,2]. This entoptic phenomenon, known as Maxwell’s spot (MS), was first described in detail by James Clerk Maxwell in 1856 [3]. Maxwell also noted similarities with the Haidinger’s brush (HB) entoptic phenomenon described several years previously [4]. The latter is seen transiently as a faint hour-glass-like pattern in central vision when viewing a uniform linearly polarized light field containing blue wavelengths [4,5,6].

Early studies [2,7,8] confirmed Maxwell’s findings and noted variability in the appearance of Maxwell’s spot ranging from a diffuse patch to an annular configuration 2–3° in diameter, with a small central spot of approximately 30′ in diameter. The boundary of the percept was variously described as smooth, ragged, circular, diamond-shaped or elliptical [2,7,8]. Unlike MS, whilst the salience of HB varies between individuals and with the orientation of incident polarization [9], the ‘brush-like’ percept remains consistent across observers [10].

The assumption that both MS and HB are dependent on macular pigments is historic [2,3,7,8]. There is substantial evidence for the involvement of macular pigments in the generation of HB [10], although plausible alternative explanations have been proposed [11]. Direct evidence for the role of macular pigments in the generation of MS is less well documented. The perceived 30′ diameter central spot in MS corresponds to the absence of S-cones in the central fovea [12,13], and is independent of macular pigments. The perceived size of MS in a small number of individuals was correlated with macular pigment distribution data derived from digital colour fundus images [13]. We recently proposed a computational model that links MS and HB as emerging from a common mechanism of differential absorption by radially symmetric deattenuating elements within the foveolar retina [6]: the principle absorbing component was assumed to be macular pigment molecules, with an overall orientation preference dictated by radial structural elements within the Henle fibre layer of the fovea.

Given this evidence, the most widely accepted hypothesis proposed for the origin of the peripheral zones in MS, and its documented perceptual variations, is absorption of blue light by macular pigments that result in a reduction of foveal photoreceptor illumination [14,15,16,17,18]. Alternative explanations not based on macular pigment include MS generation from colour contrast between a positively blue-biased parafoveal retina and a negatively blue-biased central foveola [19], and MS generation by local variations in the relative abundance of foveal photoreceptor types [1,20]. The latter is supported by the finding that macular pigment screening alone does not explain the reported effect of colour vision deficits on MS perception [1]. Thus, mechanisms of Maxwell’s spot generation separate from macular pigment absorption cannot be discounted.

Early studies on the origins of MS and HB were limited by the lack of in vivo measures of macular pigments and in vivo structural evaluation of the central macula. Of the various methods now available for assessing macular pigments in vivo [21], dual wavelength autofluorescence (DWAF) [22] is established as an objective measure of both macular pigment optical density (MPOD) and the spatial distribution of macular pigment. DWAF imagery was used to match the appearance of MS with macular pigment optical density profiles [18], supporting a role for macular pigments in the generation of MS. DWAF and other methods have demonstrated great inter-individual variations in the spatial distribution and concentration of macular pigment, both in vivo [23,24,25,26,27] and in post mortem eyes [28].

The development of optical coherence tomography (OCT) and associated technologies have revolutionized the in vivo study of the retina, and several investigations have correlated OCT-defined macular structure with macular pigment distribution and density. Correlations with MPOD include central macular thickness [29,30] and foveal width [31]. Individuals with ring profiles of macular pigment tend to have smaller distances between the inner and outer limiting membranes [32], while secondary peaks in the macular pigment spatial profile are associated with wider foveas [33], and are more likely in individuals with larger foveal avascular zones (FAZ) [34].

The aim of this study was to investigate the factors influencing the appearance of HB and MS and their reported variations, with particular reference to macular pigment and foveal anatomy. Given a reliable method for quantifying both entoptic phenomena [6], together with accurate in vivo methods for determining macular pigment distribution (DWAF) and retinal structure (OCT), the factors giving rise to MS and HB can now be investigated in greater detail than previously possible.

## 2. Materials and Methods

All measurements were completed in the School of Optometry at Aston University, UK, between January and December, 2021. The study received local ethical committee approval (Aston University Ethics Committee, #1566, 1 November 2019), and all participants gave informed consent prior to enrolment. The study conformed to the tenets of the Declaration of Helsinki. Exclusion criteria were defective colour vision and prior history or clinical evidence of ocular disease. All individuals had optimally corrected monocular visual acuities of logMAR 0.2 or better and underwent a slit-lamp biomicroscopic examination that included indirect ophthalmoscopy. Strict COVID-19 precautions, including the use of personal protective equipment, were observed in those parts of the study taking place during the pandemic.

Monocular measurements from 52 eyes were made from a participant pool of 13 males and 13 females (age range 28 to 64 years; mean age ± standard deviation = 46.2 ± 11.7 years).

The study comprised two experiments. Experiment 1 compared the measured horizontal extent of MS and HB with that of DWAF-derived macular pigment measurements and OCT-defined anatomical features of the fovea and foveola. Experiment 2 compared the subjective appearance of MS with the distribution pattern of macular pigment for the individual eyes of each participant.

**Figure 1:**
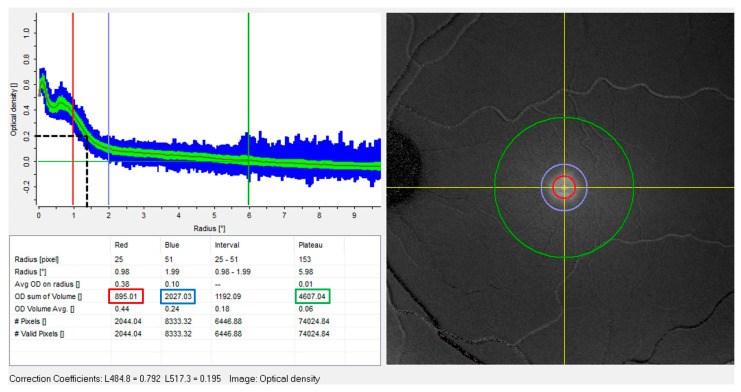
Machine−generated output of macular pigment optical density distribution analysis for case 16 (category 2; central peak and ring). The right image is a digital subtraction of green− and blue−light autofluorescence data, centred on the foveola (intersection of yellow axes) with 1° (red), 2° (blue) and 6° (green) radius circles. Top left is a graph of MPOD (vertical axis) along circular paths of radii given on the horizontal axis. The blue zone spans the maximum and minimum optical density at the given radius, the green zone spans the standard deviation and the continuous black curve is the mean optical density at this radius. The black dashed horizontal/vertical lines indicate the measurement of the radius at which the mean macular pigment optical density has a value of 0.2 (MPr0.2). The table shows data for 1° (red), 2° (blue) and 6° (green) circular paths. In all cases the 6° path is taken as the zero datum for optical density. Red, blue and green boxes indicate sum of macular pigment volume within 1° (MPV1), 2° (MPV2) and 6° (MPV6) radii, respectively (see Table 1).

Measurement of macular pigment density was performed using a Heidelberg Spectralis OCT/SLO (Heidelberg Engineering GmbH, Heidelberg, Germany) with DWAF capability, and the investigational MPOD module. Pupils were dilated prior to macular pigment measures according to standard clinical protocols, and the MPOD method followed that detailed elsewhere [35]. For each participant, machine-generated blue (488 nm) and green (514 nm) light autofluorescence images were grabbed for processing according to the manufacturer’s methodology. MPOD data was taken from the machine output (Figure 1), as described elsewhere [31,36], and comprised averaged MPOD values and MPOD volume measures along and within circular paths with radii from the foveal centre of 0.2°, 1°, 2° and at a reference radius of 6° (corresponding to machine values of 0.20°, 0.98°, 1.99° and 5.98°). The machine-generated macular pigment metric used in the present study was the volume sum within the four circular paths (‘OD sum of volume’). The radius at which the MPOD value equaled 0.2 (MPr0.2) was measured from the machine-generated MOPD data output (Figure 1, left upper panel) using ImageJ image analysis software [37]. The MPr0.2 measure, devised as an index of the radial extent of macular pigment, coincides with the exponentially declining part of the macular pigment spatial distribution.

**Figure 2:**
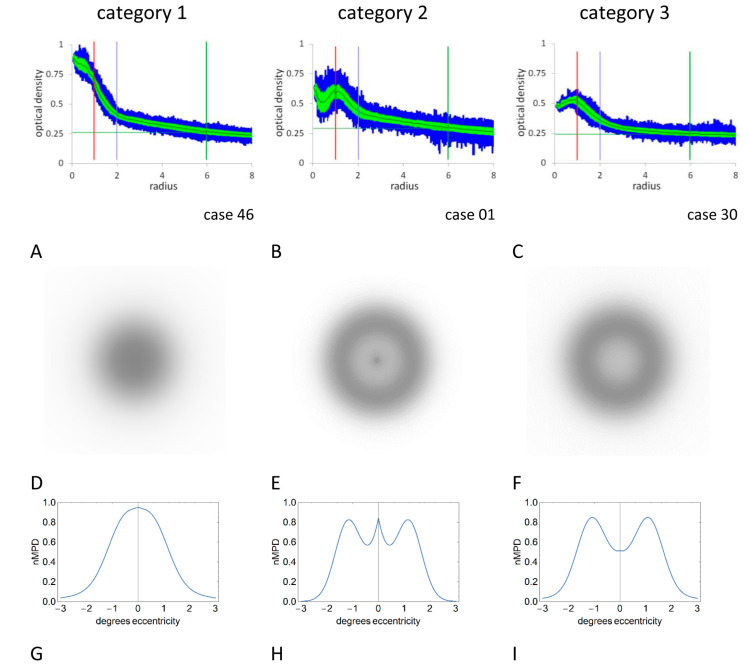
Macular pigment optical density profiles (**A**–**C**), two dimensional macular pigment density/MS simulations (**D**–**F**) and normalized one-dimensional macular pigment density profile simulations (nMPD, (**G**–**I**)). Profile categories 1 (**A**,**D**,**G**), 2 (**B**,**E**,**H**) and 3 (**C**,**F**,**I**) are described in the text. See Figure 1 for explanations of macular pigment spatial profiles. Images (**D**–**F**) were used in Experiment 2.

The macular pigment profiles were classified blind of experimental results independently by authors GM and SA into one of four categories that summarized the main macular pigment distribution morphologies [26,27] (Figure 2A–C). There was agreement between the two assessors for all cases, apart from two when a consensus was reached after discussion. Category 1 had a central peak with a generally monotonic decline; category 2 had a central peak with secondary annular peak, followed by a monotonic decline; category 3 had a central trough with an annular peripheral peak, followed by a monotonic decline. Absence of an identifiable MPOD profile was classified as category 0.

**Figure 3:**
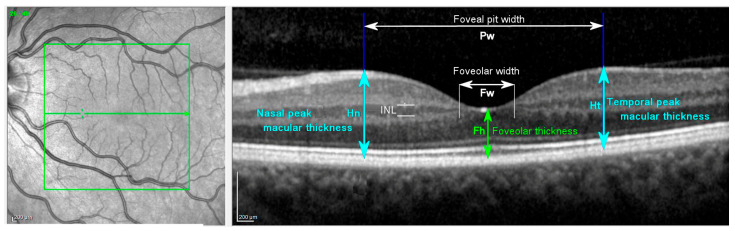
Definitions of foveal and foveolar morphology. Participant 41 left eye. Left panel is an infrared scanning laser ophthalmoscope fundus image with a superimposed 20° square box and bisecting horizontal arrow that runs through the foveolar centre. Right panel is the OCT image scanned along the horizontal arrow in the SLO image. Annotations are: INL, inner nuclear layer of retina (section between horizontal thin white lines); Fh foveolar thickness (central macular thickness, light green); Fw foveolar width (white horizontal arrow) is the distance between adjacent terminations of the INL (thin vertical white lines); Hn, nasal peak macular thickness (light blue); Ht temporal peak macular thickness (light blue); Pw, foveal pit width. The foveal pit height (Ph) is calculated as the difference between Fh and the average of Hn and Ht. The foveolar radius (Fr) and foveal pit radius (Pr) are half the respective widths measured in degrees of visual angle. Scale bars are in μm (machine values). The horizontal width of the OCT scan is 20° of visual angle.

Foveal morphometric data was obtained either as machine measures or from manual morphometry of high resolution spectral-domain OCT images presented as 20° × 20° blocks using the Heidelberg Spectralis OCT facility. Apart from machine-derived measures, quantification of foveal and foveolar morphology was obtained using imageJ and machine-generated images (Table 1, Figure 3). The foveolar boundary was defined by the termination of the inner nuclear layer (INL in Figure 3), and the foveolar radius (Fr) was defined as half the distance between adjacent terminations of the INL in the horizontal plane OCT (Fw in Figure 3). The boundary of the foveal pit was defined as the peak thickness of the perifoveolar neuroretina [38], and the foveal pit radius (Pr) was defined as half the distance between the maximum nasal and maximum temporal macular thicknesses in the horizontal plane OCT (Pw, Hn, Ht in Figure 3). Linear measurement data in the plane of the retina were recorded as degrees of visual angle for data consistency, and to avoid confounding errors due to magnification effects from inter-individual variations in ocular dimensions [39]. Machine-generated axial measures of retinal thicknesses within the region investigated were assumed to be consistent between cases and were expressed in μm.

Experiment 1 required measurement of the horizontal width of MS and HB, as detailed elsewhere [6]. In brief, the apparatus consisted of a diffused unpolarized light source of red (633 nm), green (519 nm) and blue (456 nm) light emitting diodes (LED), viewed through a Maxwellian system with a micrometer eyepiece.

MS was observed by alternating the LED illumination between combinations of red and blue, and red and green, at 1 Hz. MS was perceived with the red/blue combination as a reddish spot against a purple background (see Graphic Abstract left panel for simulation). Using a combination of red and green light, either MS was absent or an after image was perceived against an orange/yellow background.

HB was generated by inserting a liquid crystal linear polarization rotator into the optical system, with constant blue/red illumination. Alternating the axis of linear polarization from horizontal to vertical at a rate of 2 Hz produced a corresponding and persistent HB percept in most observers (see Graphic Abstract right panel for simulation). The choice of optimum modulating frequencies for both wavelength and polarization changes was determined empirically, as detailed elsewhere [6].

The task of the observer was to adjust the micrometer caliper to the perceived horizontal width of either MS or HB. Three measures were completed for each entoptic phenomenon, and the averaged micrometer setting was converted into a visual angle following appropriate calibration. The width measure was halved to give a radius compatible with the macular pigment data. Whilst the ‘brush’ configuration of HB was reported in all cases where the phenomenon was perceived, variability in the appearance of MS was noted. All participants were able to set the caliper to the perceived boundaries of both phenomena.

Experiment 2 required identification of the perceived appearance of MS (MS perceptual profile). MS was generated using a light box comprising a light diffusing filter placed between the eye and an LED array with the same characteristics employed for the micrometer measures. The box was held at normal reading distance and observers were asked if they could perceive MS with each eye separately. The perceived image was compared with three simulated images on a test chart (Figure 2D–F). The test images were a 2-dimensional representation of mathematically modeled macular pigment spatial profiles (Figure 2G–I), and are idealised representations of the pigment spatial distribution variants described above [40]. The monocular responses were assigned a score of 0 (i.e., not seen), 1, 2 or 3 depending on which simulation category most closely resembled the individual’s percept. In order to allow direct comparison with previous studies, categories 2 and 3 were grouped into a ‘ring’ category for subsequent analysis and comparison with the ‘spot’ category 1.

Conventional parametric and non-parametric statistical methods were used when appropriate. Independent two-sample t-tests were used unless otherwise stated. Correlations of normal parametric data were completed with Pearson’s product moment coefficient (R). Multiple regression analysis was performed by conventional and stepwise forward methods. Statistical significance was assumed for *p* < 0.05. Fleiss’ kappa statistic (κ) was used to determine agreement of outcome of Experiment 2 data, with ‘good’ agreement between methods being defined as 0.6 ≤ κ ≤ 0.8 and ‘very good’ agreement defined as κ > 0.8 [41].

## 3. Results

Data sets were available for 49 healthy eyes from 26 participants. Five data sets were incomplete for Experiment 1, and 8 data sets were incomplete for Experiment 2. Only cases with complete data for all variables were included in correlation/regression analyses. Different components of the study were performed on different occasions, with incomplete data sets resulting from participant unavailability.

Macular pigment and foveal morphometric OCT data are summarized in Table 1 and Table 2. Of the 49 macular pigment optical density spatial profiles, 26 were classified by the assessors as category 1 (‘spot’), 18 as category 2 and five as category 3 (i.e., there were 23 eyes in the ‘ring’ category). There were no appreciable gender-dependent differences (category 1: M = F =13; category 2 + 3: M = 12, F = 11).

Compared with the spot category, foveolar thickness was significantly less in the ring category (spot Ft mean 239.2 μm; ring Ft mean 219.3 μm; *p* < 0.01), while both foveolar radius (spot Fr mean 0.76°; ring Fr mean 0.87°; *p* = 0.01) and foveal pit height (spot Ph mean110.9 μm; ring Ph mean 137.2 μm; *p* < 0.01) were significantly greater. There was no significant difference in foveal pit radius between the spot and ring categories. Central macular pigment optical density was significantly lower in the ring compared to the spot category (spot MPVc mean 64.4; ring MPVc mean 48.4; *p* < 0.01), consistent with the definitions of the two categories. There were no significant differences in other macular pigment parameters.

### 3.1. Experiment 1: Horizontal Radius of MS and HB, Macular Pigment Density and OCT Morphometry

Summary statistics are given in Table 1 and Table 2. MS subtended a larger visual angle (mean MSr = 1.4°) than that of the anatomically defined foveola (mean Fr = 0.81°), but a smaller visual angle than the boundary of the foveal pit (Pr = 3.96°). The differences were significant in all cases (*p* < 0.05). There was a significant difference (*p* = 0.02) in MS radius between the spot (category 1) and ring categories (categories 2 + 3).

While the radius of HB was significantly greater than that of MS (mean HBr = 1.6°, *p* = 0.03), there was a significant linear correlation between the radii of the two entoptic phenomena (HBr v MSr, R^2^ = 0.38, *p* < 0.05, HBr = 0.85.MSr + 0.49). Haidinger’s brushes were significantly larger in ring (mean HBr = 1.9°) compared to spot (mean HBr = 1.4°, *p* < 0.05) macular pigment density profiles.

Bivariate regression analyses were performed for MSr and HBr (Y variables), and the nine macular pigment/foveolar measurements (X variables). There was a high degree of X variable intercorrelation within each macular pigment and foveolar measurement group, with less intercorrelation between groups (Supplementary Table S1).

Conventional multiple regression analysis (Supplementary Table S2) established a linear regression of Y variables in relation to all nine X variables and estimated the β (slopes) of each variable. Significant multiple linear regressions were fitted to both MS and HB radii. The MP variable MPr0.2 (β = 1.56) was significantly related to MSr (R^2^ = 0.60 *p* < 0.001), and the morphometric measurements Ft (β = 0.55), Fr (β = 0.47), and Ph (β = 0.62) were significantly related to HBr (R^2^ = 0.72 *p* < 0.001).

Stepwise multiple regression by the ‘forward method’ (Supplementary Table S3) identified those variables significantly related to Y and ranked them in order of importance. The analysis selected two variables significantly associated with MSr: MPr0.2 which accounted for approximately 47% of the total variance, and MPV2 which accounted for 8% of the remaining variance. For HBr, there were four significantly associated variables: MPr0.2, Fr, Pr and Ft, which accounted for approximately 36%, 19%, 6% and 7% of the total variance, respectively. Note that Ph was not selected by stepwise multiple regression analysis.

Differences in the details of the two multiple regression methods relate to differences in the models used for selecting variables, significance testing, and the different effects of the degree of intercorrelation among the variables.

### 3.2. Experiment 2: Light Box Generation of MS

Complete data sets for macular pigment spatial profiles and MS perceptual profiles were available for 41 eyes (Table 3 and Table 4). No MS percept (category 0) was recorded for one eye, category 1 MS perceptual profiles (‘spot’) were recorded for 16 eyes, category 2 profiles were recorded for 11 eyes and category 3 profiles were recorded for 13 eyes (i.e., 24 eyes were classified as ‘ring’ perceptual profiles).

When classified into spot (category 1) and ring (categories 2 + 3) categories, there was good agreement (κ = 0.90, 95% CI 0.61 to 1.00, *p* < 0.001) between MS perceptual profile and macular pigment distribution profile, with equivalence of categories in 39 of 41 eyes tested. Furthermore, the macular pigment and OCT results for the spot/ring classification of MS perceptual profile and pigment spatial distribution are similar (compare Table 3 with Table 2). In particular, for both MS perceptual profile and macular pigment distributions, ring distributions had significantly thinner foveolar thicknesses (Ft) and deeper foveal pit heights (Ph). For MS but not macular pigment, a ring distribution had a significantly wider foveal pit radius (Pr), whereas for macular pigment but not MS, a ring distribution has a significantly wider foveolar radius (Fr).

When the ring category was subdivided into ring with central peak (category 2) and ring only (category 3), the correspondence remained (Table 4) with good agreement between the observed and measured categories (κ = 0.74, 95% CI 0.53 to 0.96, *p* < 0.001). The greatest non-correspondence (*n* = 5) occurred in MS perceptual profile categories 2 and 3 for eyes with category 2 macular pigment density profiles (ring and central peak).

Unlike the macular pigment density profiles, there was no significant difference in MS radius between the spot and ring perceptual profile categories. The difference in HB radius between the two categories was significant (*p* = 0.04).

## 4. Discussion

Multiple regression analysis of Experiment 1 data identified two important differences between the associations of MS and HB size: (i) the radius of MS is associated with macular pigment variables (MPr0.2, MPV2) but not foveolar measurement variables; and (ii) the radius of HB is associated with foveolar measurement variables (Ft, Ph, Pr) but not macular pigment variables, apart from MPr0.2. The correlation of both MS and HB radii with a radius measure of macular pigment optical density explains, at least in part, the correlation between MS and HB radii.

The association between MS and macular pigment was further reinforced by the results of experiment 2. The spatial density pattern of macular pigment closely matched the subjective appearance of MS (excluding the central S-cone scotoma), supporting the hypothesis that variations in macular pigment patterns are responsible for variations in perceived MS morphology. This result is compatible with those of Delori et al., who reported a qualitative match between the appearance of MS and macular pigment optical density profiles determined by DWAF imaging [18]. Furthermore, and consistent with previous reports [32,33], individuals reporting a ring perceptual profile had thinner foveolas, with deeper and wider foveal pits compared with those perceiving a spot profile.

In other respects, there is conformity of the results of this study with those published elsewhere. The measured horizontal extent of MS (mean diameter 2.8°, range 0.4–4.6°) and HB (mean diameter 3.2°, range 0.0–6.0°) are consistent with previous findings of mean diameters of approximately 3° [1,2] for MS and 5° for HB [10,42]. The difference in size between MS and HB was significant and confirms previous comparisons [6]. Both MS and HB radii fall between the anatomically defined boundaries of the foveola and the perimeter of the foveolar pit. This is consistent with the accepted location of the site of generation of luminance signal of MS and HB to the Henle fibre layer [10], which forms the non-photoreceptor component of the foveolar retina and which attenuates centrifugally between the boundaries of the foveola and fovea.

Other results consistent with previous findings include correlations between foveolar thickness (Ft, central retinal thickness) with central macular pigment densities (MPVc and MPV1) [29,30]. We were unable to demonstrate a correlation between macular pigment parameters and foveal width [31].

This study quantitatively relates variations in the appearance of MS and HB to variations in macular pigment distribution patterns in support of previous findings [10,13,18,43]. Our novel findings of the different associations of MS and HB indicate that HB (as measured by its horizontal radius) is also dependent on foveolar structural variables. This was not the case for the radius of MS which had no significant association with foveolar structural variables. Such findings may account, at least to some extent, for the observed differences in the sizes of MS and HB. Whilst high values of corneal retardation affect the contrast of HB [9,44], the effect is small in most individuals [40]. The effect of corneal retardation is further minimized by measuring HB close to an axis of retardation [43,44] such as the horizontal meridian, as in this study. The different associations of MS compared to HB have implications regarding potential applications of the phenomena. Whilst measures of MS dimensions directly reflect macular pigment density and distribution, measures of HB might give a more general measure of foveal functional dependence on both macular pigment density and foveal structural integrity.

The findings of this study relate only to radius measures of MS and HB. Other psychophysical measures of MS and HB, currently under investigation, may show different associations. Furthermore, and despite the strong evidence that macular pigment pre-receptoral screening is a sufficient mechanism for MS, the reported variations in MS perception in colour-defective individuals [1,20] requires investigation, as does the perception of HB in these individuals.

Rather than dismissing non-macular pigment-based theories of generation our findings and those of others suggest that co-mechanisms of HB and, to a lesser extent, MS cannot be excluded. In particular, further study is required to determine the relationship between MS and HB perception and foveal structure, and how both phenomena relate to photoreceptor variability [1].

## 5. Conclusions

The results of this study support the theory that the principal mechanism of MS generation is pre-receptoral screening by macular pigment. Whilst macular pigment plays a role in the perception of HB, additional factors relating to foveal structure are also relevant.

## Figures and Tables

**Table 1:** Measured parameters: definitions and basic statistics for all cases (i.e., sum of all three categories).

Parameter	Description	n	Min	Max	Mean	sd
MSr	Maxwell spot radius (°)	49	0.2	2.3	1.4	0.5
HBr	Haidinger brush radius (°)	48	0.0	3.0	1.6	0.7
MPVc	central macular pigment volume (arbitrary units)	49	25	101	56.9	19.0
MPV1	sum of macular pigment volume within 1° radius (arbitrary units)	49	408	1629	918.9	331.0
MPV2	sum of macular pigment volume within 2° radius (arbitrary units)	49	846	3843	2172.1	823.7
MPV6	sum of macular pigment volume within 6° radius (arbitrary units)	49	1581	9500	5086.4	1983.9
MPr0.2	radius at which MPOD = 0.2 (°)	49	0.60	2.23	1.50	0.41
Ft	foveolar thickness (μm)	49	199	272	229.9	15.1
Fr	foveolar radius (°)	49	0.51	1.13	0.81	0.16
Ph	foveal pit height (μm)	49	68	166	123.2	21.4
Pr	foveal pit radius (°)	49	2.80	5.09	3.96	0.44

**Table 2:** Results for cases categorized into spot (category 1) and ring macular pigment density profile categories (categories 2 + 3). The final column contains *p*-values for 2-tailed independent two-sample t-tests comparing the given parameter in spot and ring macular pigment categories.

		Spot Density Profile					Ring Density Profile					
Parameter		n	Min	Max	Mean	sd	n	Min	Max	Mean	sd	*p*
Maxwell spot radius	MSr	24	0.2	2.1	1.2	0.5	23	0.5	2.3	1.5	0.5	0.020 *
Haidinger brush radius	HBr	23	0.0	2.3	1.5	0.8	23	1.1	3.0	1.9	0.6	0.004 *
Macular pigment optical density												
MPVc		26	25	101	64.4	22.2	23	29	64	48.4	9.2	0.002 *
MPV1		26	408	1629	971.2	421.4	23	452	1142	859.7	174.3	0.225
MPV2		26	846	3843	2165.4	1005.2	23	1116	3278	2179.8	576.4	0.951
MPV6		26	1581	9500	5188.7	2359.3	23	2630	7143	4970.7	1495.2	0.698
MPr0.2		26	0.60	2.23	1.43	0.48	23	0.77	2.10	1.57	0.32	0.229
OCT morphometry												
Foveolar thickness	Ft	26	199	272	239.2	14.2	23	207	232	219.3	7.2	<0.001 *
Foveolar radius	Fr	26	0.51	1.06	0.76	0.17	23	0.67	1.13	0.87	0.13	0.010 *
Foveal pit height	Ph	26	68	155	110.9	17.4	23	113	166	137.2	16.5	<0.001 *
Foveal pit radius	Pr	26	3.38	4.72	3.87	0.35	23	2.80	5.09	4.06	0.51	0.126

* *p* < 0.05.

**Table 3:** Macular pigment and OCT results for MS classified into spot (category 1) and ring (categories 2 + 3) perceptual profile configurations. Final column (*p* MS) shows results of a paired t-test comparing the spot/ring groups for each parameter. Other abbreviations as in Table 1.

		Spot Perceptual Profile					Ring Perceptual Profile					
Parameter		n	Min	Max	Mean	sd	n	Min	Max	Mean	sd	*p*
Maxwell spot radius	MSr	14	0.2	2.1	1.3	0.6	24	0.5	2.3	1.6	0.5	0.136
Haidinger brush radius	HBr	14	0.0	2.3	1.4	0.9	25	0.6	3.0	1.8	0.6	0.039 *
Macular pigment optical density												
MPVc		16	25	101	60.3	24.1	24	29	78	50.7	12.2	0.156
MPV1		16	408	1620	897.5	445.8	24	629	1173	898.6	168.9	0.992
MPV2		16	846	3843	2034.9	1082.1	24	1423	3278	2247.5	538.0	0.475
MPV6		16	1581	8800	5028.5	2400.4	24	2630	7143	5089. 7	1438.1	0.928
MPr0.2		16	0.60	2.15	1.36	0.52	24	1.25	2.10	1.61	0.27	0.091
OCT morphometry												
Foveolar thickness	Ft	16	199	258	237.0	14.0	24	207	254	223.9	11.8	0.006 *
Foveolar radius	Fr	16	0.51	1.06	0.77	0.18	24	0.61	1.13	0.87	0.15	0.092
Foveal pit height	Ph	16	87	134	109.6	12.8	24	94	166	133.3	18.3	<0.001 *
Foveal pit radius	Pr	16	3.48	4.35	3.79	0.26	24	2.80	5.09	4.09	0.51	0.017 *

* *p* < 0.05.

**Table 4:** Light box results. Cell values are numbers of eyes. Rows are chart categories, and columns are MPOD categories.

		MPOD Category				
		0	1	2	3	Sum
**Chart category**	**0**	0	1	0	0	1
	**1**	0	16	0	0	16
	**2**	0	0	11	0	11
	**3**	0	1	5	7	13
	**Sum**	0	18	16	7	41

## Data Availability

Data is contained within the article and Supplementary Material.

## Supplementary Materials

The following supporting information can be downloaded at: https://www.mdpi.com/article/10.3390/vision7010011/s1, Table S1: Pearson correlation coefficients of macular pigment/foveolar measurements, Table S2: Multiple regression (conventional method), Table S3: Multiple regression (stepwise forward method).
