# Supplementary material for: The Differential Contribution of Macular Pigments and Foveal Anatomy to the Perception of Maxwell’s Spot and Haidinger’s Brushes
Source: Vision (Basel). 2023 Feb 6;7(1):11. doi: 10.3390/vision7010011 (PMC9944110; doi:10.3390/vision7010011)
Supplement: Supplementary file 1 [file vision-07-00011-s001.zip › vision-2043781-supplementary.pdf]

The differential contribution of macular pigments and foveal anatomy to the perception of Maxwell's spot and Haidinger's brushes

Gary P Misson, Rebekka Heitmar, Richard Armstrong and Stephen J Anderson.

Supplementary Tables

Table S1. Pearson correlation coefficients of macular pigment / foveolar measurements (X variables). Parameter definitions as in Table 1. Values with a significance level of $p < 0.05$ are underlined. Note high degree of intercorrelation within each macular pigment and foveolar measurement group (shaded areas) with less intercorrelation between groups (boxed area).

	MPVc	MPV1	MPV2	MPV6	MPr0.2	Ft	Fr	Ph
MPV1	0.85							
MPV2	0.69	0.94						
MPV6	0.68	0.9	0.95					
MPr0.2	0.57	0.86	0.96	0.91				
Ft	0.52	0.28	0.09	0.08	-0.02			
Fr	-0.24	-0.03	0.13	0.04	0.22	-0.52		
Ph	-0.36	-0.06	0.19	0.13	0.3	-0.73	0.52	
Pr	-0.04	0.08	0.06	0.04	0.09	-0.32	0.24	0.13

Table S2. Multiple regression (conventional method).

Y	R	R^2	F	DF	p
**MSr**	0.78	0.60	6.19	10,37	<0.000
(Significant X variables: MPr0.2 β = 1.56)					
**HBr**	0.85	0.72	10.04	9.36	<0.000
(significant X variables: Ft, β = 0.546; Fr, β = 0.470; Ph, β = 0.618)					

Table S3. Multiple regression (stepwise forward method).

Y variable	X selected	Rank	Multiple R	R^2	R^2 change	F to enter/exclude	p
MSr	MPr0.2	1	0.69	0.47	0.47	38.9	<0.000
	MPV2	2	0.74	0.55	0.08	7.3	<0.009
HBr	MPr0.2	1	0.60	0.36	0.36	24. 9	<0.000
	Fr	2	0.74	0.55	0.19	17.7	<0.000
	Pr	3	0.78	0.61	0.06	6.4	0.015
	Ft	4	0.84	0.70	0.07	9.2	0.004
